# How mHealth Can Contribute to Improving the Continuum of Care: A Scoping Review Approach to the Case of Human Immunodeficiency Virus in Sub-Saharan Africa

**DOI:** 10.3389/phrs.2022.1604557

**Published:** 2022-09-23

**Authors:** Philippe Lepere, Awa Babington-Ashaye, Guillermo Z. Martínez-Pérez, Didier Koumavi Ekouevi, Alain Bernard Labrique, Alexandra Calmy

**Affiliations:** ^1^ Institute of Global Health, Faculty of Medicine, University of Geneva, Geneva, Switzerland; ^2^ FIND, The Global Foundation for Diagnostics, Geneva, Switzerland; ^3^ Département de Santé Publique, Faculté des Sciences de la Santé, Université de Lomé, Lomé, Togo; ^4^ INSERM U1219 Bordeaux Population Health Centre Recherche (BPH), Bordeaux, France; ^5^ Department of Epidemiology, Johns Hopkins Bloomberg School of Public Health, Baltimore, MD, United States; ^6^ Department of International Health, Johns Hopkins Bloomberg School of Public Health & Johns Hopkins University Global Digital Health Initiative, Baltimore, MD, United States; ^7^ Service des Maladies Infectieuses, Hôpitaux Universitaires de Genève (HUG), Geneva, Switzerland

**Keywords:** sub-Saharan Africa, HIV, mHealth, continuum of care, patient-healthcare provider

## Abstract

**Objectives:** To determine mHealth’s contribution to improving the continuum of care in sub-Saharan Africa towards achieving treatment targets for human immunodeficiency virus (HIV) endorsed by the 2016 Political Declaration on ending acquired immunodeficiency syndrome (AIDS).

**Methods:** PubMed, Medline, Embase, Web of Science Core Collection and Cochrane databases; three observatories and four repositories were searched to identify and select relevant articles, projects and guidelines published from 1 January 2017, to 30 April 2021. Records focusing on the use of mHealth related to HIV treatment cascade or healthcare provider/patient relationship were considered.

**Results:** From 574 identified records, 381 (206 scientific manuscripts and 175 mHealth projects) were considered. After screening, 36 articles (nine randomized control trials, five cohort studies, 19 qualitative studies, and three economic studies) and 23 projects were included.

**Conclusion:** The cross-cutting benefits of mHealth that enhance patient empowerment have been identified. Important challenges such as gaps between research and implementation, lack of transdisciplinary collaboration, and lack of economic evidence were identified to support future mHealth research and accelerate the achievement of treatment targets for HIV.

## Introduction

With the aim of ending acquired immunodeficiency syndrome (AIDS) as a public health threat by 2030, a political declaration was adopted at the United Nations High Level Meeting on Ending AIDS in 2016,^1^ including a set of specific, time-bound targets to be reached. By 2020, 90% of the people living with HIV (PLHIV) are expected to know their HIV status, 90% of those diagnose with HIV infection receive sustained antiretroviral therapy (ART), and 90% of those receiving ART have durable viral suppression. The target for 2025 is to reach 95-95-95 for each stage. In addition, Lazarus et al. proposed an additional target to ensure that 90% of the people with viral load suppression have a good health-related quality of life [1].

Digital health is defined as the “use of information and communications technologies (ICT)” for development “in support of health and health-related fields, including healthcare services, health surveillance, literature, and education; knowledge and research” [2]. It can be used for a wide range of purposes, including health promotion and illness prevention, healthcare delivery, training and supervision, electronic payments, and access to medical information systems. Mobile health (mHealth) is a component of digital health that uses mobile devices and connectivity.

Since 2011, the World Health Organization (WHO) has promoted the use of innovative mobile technologies to overcome barriers that undermine access to healthcare and the quality of care delivery in low- and middle-income countries (LMICs) [3]. In 2012, a Cochrane review concluded that weekly mobile phone text messaging could be effective in enhancing adherence to ART [4]. In 2013, the WHO endorsed text messaging or short messaging service (SMS) interventions to support individual-level adherence to ART and improve the linkage of people diagnosed with HIV to HIV care services [5]. Finally, a 2016 report by the WHO Global Observatory for eHealth indicated that universal health coverage cannot be achieved without the support of mHealth [6].

There were nearly 5.27 billion unique mobile phone subscribers at the end of 2021 and the global penetration rate stood at 67%, with regional disparities ranging from 86% in Europe to 46% in Sub-Saharan Africa (SSA) [7]. The mobile monthly data traffic stood at 11.4 GB globally and 2.9% GB in SSA [7].

Considering these data and the improving ecosystem in many SSA countries, as well as systemic bottlenecks hindering the AIDS response, the assumption was made that the use of mobile phones could contribute to improving outcomes throughout the various stages of the HIV treatment cascade towards achieving both the 90-90-90 targets and the “fourth 90.”

The primary objective of this review was to provide a global overview and map of existing scientific studies, implemented projects, and available tools and guidelines for SSA that show how mHealth can contribute to improving the continuum of care towards achieving the HIV treatment targets. Secondary objectives included 1) understanding mHealth-related challenges encountered by the researchers during the conduct of their studies and 2) identifying lessons learned to advance future mHealth strategies and policies within the framework of global health.

The scoping review methodology was selected as the most appropriate tool for responding to review objectives because 1) the review required considering findings from a body of knowledge that is heterogeneous in methodology, sample size, primary outcomes and mHealth tool properties; 2) the goal was not to discuss the quality of published studies nor to perform an assessment of methodological limitations or risk of bias, but rather to map emerging evidence and identify key research gaps and priorities to advance future mHealth strategies and policies; and 3) the willingness to integrate a wide range of data sources beyond scientific publications (with gray literature, such as reports, tools, and guidelines, that are considered relevant sources of data) [8].

By setting up the date of 1 January 2017, for article selection, the review was positioned at the crossroads between the key turning point in the AIDS response in 2016 and the promotion of use of mHealth solutions by the WHO to improve health outcomes.

## Methods

### Study Definition

Arksey and O’Malley’s definition of a scoping review and the six-stage framework for conducting such a review were adopted: 1) identifying the research question, 2) searching for relevant studies, 3) selecting studies, 4) charting the data, 5) collating, summarizing and reporting the results, and 6) consulting with stakeholders to inform or validate study findings [9].

The research question was defined as follows: “Can mHealth contribute to improving the continuum of care towards achieving the 95-95-95 HIV treatment target by 2025 in SSA?” This review question implied mapping of existing studies, projects, and guidelines that have demonstrated a noticeable interest in mHealth, contributed to improving one or more stages of the HIV treatment cascade in SSA, or have shown a lack of effectiveness. This review question also helped identify research gaps.

### Inclusion Criteria

Articles were eligible for inclusion if they: 1) involved a qualitative study, quantitative studies such as cohort studies, randomized control trials or economic study using mHealth solutions for mobile phones, either as a component of or as a stand-alone research project; 2) reported an outcome from the use of mobile phones related to one or more steps of the treatment cascade: uptake of HIV testing, linkage to HIV care, uptake of ART, retention in care, viral load monitoring, transmission of laboratory results; 3) were available in English or French languages; 4) were published between 1 January 2017 and 30 April 2021; 5) were carried out in SSA. Study protocols, review articles, and articles that were not open access or accessible through the University of Geneva and the Swiss Library Service Platform Swisscovery were excluded. Articles promoting the use of tablets, social media, and other digital health tools that are neither easily accessible (due to network connection and digital literacy) nor affordable in many parts of Africa were excluded. Articles were also excluded if they 1) focused on HIV prevention, including behavior change, prevention of mother-to-child HIV transmission (PMTCT) and early infant diagnosis (EID); 2) aimed at reinforcing the health system or community system, or at equipping community health workers with devices to remove structural barriers; and 3) conducted before the country adopted the “test and treat” strategy promoted by the WHO in 2016, unless the authors addressed the issue of CD4 count and the potential consequences for treatment initiation and use of mHealth in their discussion [5].

### Search Strategy

PubMed, Medline, Embase, the Web of Science Core Collection and Cochrane databases were searched using keywords with the Boolean operators AND and OR. A detailed search strategy is presented in Supplementary File S1.

### Review of Articles

All articles were screened by the first author, who examined the titles and abstracts to accept or reject each article for full-text review. Subsequently, a full-text review was conducted to assess the eligibility. The co-authors supported the first author regarding the rationale and relevance of the screened studies. Furthermore, all extracted data were shared for verification by other authors.

### Data Extraction

**FIGURE 1:**
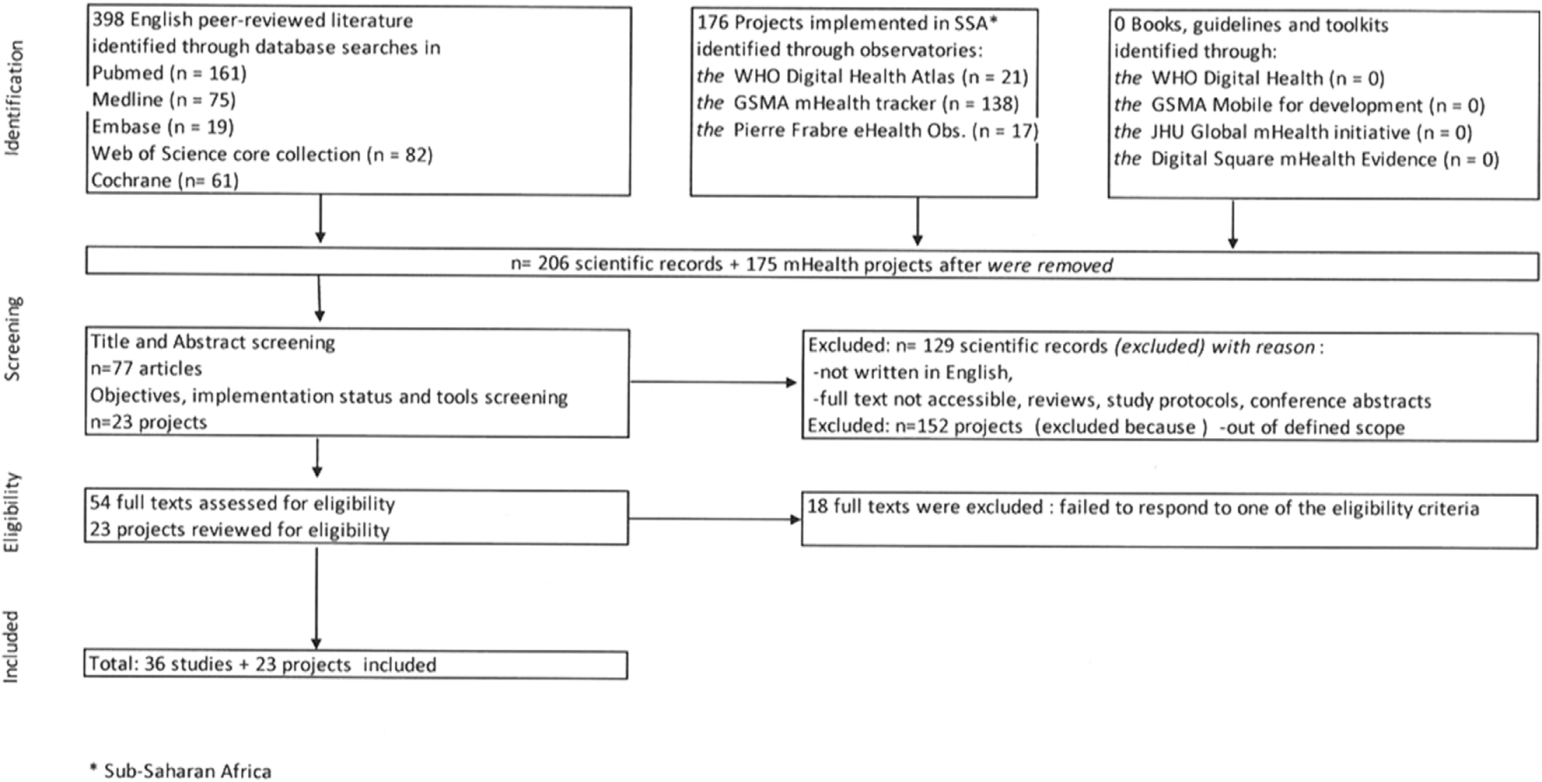
Flowchart of articles selection process (scoping review, Sub-Saharan Africa, 2017–2021).

EndNote 20 software was used to import all the selected studies from the different databases [10]. First, all identified duplicates were eliminated, and then a search for duplicates based on titles and authors’ names was conducted. Data were charted using the Preferred Reporting Items for Systematic Reviews and Meta-Analyses (PRISMA) approach [11] (Figure 1) and its extension for scoping reviews PRISMA-ScR [12] (Supplementary File S2).

**FIGURE 2:**
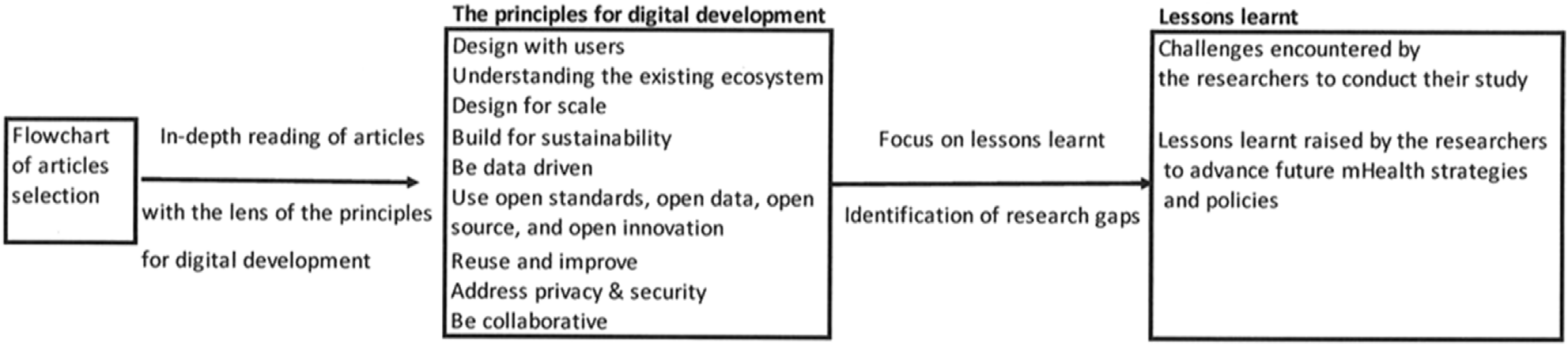
Identification of challenges raised by the researchers related to the principles for digital development (scoping review, Sub-Saharan Africa, 2017–2021).

The extraction criteria included objectives, study design, sample size, type and duration of interventions, outcome measures, and findings. The extracted data were reported using an MS Excel spreadsheet with the following information: first author, date of publication, journal, location, study design, sample characteristics, tool properties, goal of the intervention, outcomes, and results (Supplementary File S3). Subsequently, reference was made to Resolution WHA 71.12.4, endorsed by the World Health Assembly in May 2018 [13]. Recognizing that the WHO recommends any digital health solution to be developed and implemented in accordance with the principles of digital development,^2^ a deep dive into each identified article was undertaken [14] (Figure 2).

### Review of Grey Literature

Repositories of mHealth initiatives provided by the WHO Digital Health Atlas,^3^ Global System for Mobile Communications Association (GSMA) mHealth Tracker tool,^4^ and Pierre Fabre Foundation’s eHealth Observatory^5^ were also considered. Projects that 1) aim to use mHealth tools to improve uptake of HIV testing, linkage to HIV care, uptake of ART, retention in care, viral load monitoring, transmission of laboratory results, or the healthcare provider/patient relationship; 2) were implemented by governments, non-governmental organizations, or technical and financial partners; and 3) were being carried out in SSA were included. Projects focusing on HIV prevention, including behavior change, PMTCT, and EID, as well as those that were implemented before the country adopted the “test and treat” strategy, were excluded. Projects based on device distribution were also excluded because such distribution strategies might not be easily scalable countrywide. The extraction criteria included objectives, sample size, type and duration of interventions, reported outcome, and findings. Relevant information was reported in an MS Excel spreadsheet (Supplementary File S4) with a focus on the name of the project, its location, the implementing organization, tool properties, partnership, goal of the intervention, nature of interventions, and results.

Finally, a search was conducted on dedicated web sites, such as the WHO Global Observatory for eHealth,^6^ WHO Digital Health,^7^ Digital Square Resources Library/mHealth Evidence,^8^ and John Hopkins University/Global mHealth Initiative^9^ to identify tools and technical or strategic guidelines published from 1 January 2017 to 30 April 2021.

## Results

### Description of Eligible Scientific Studies

A total of 398 scientific records were identified. After duplicates were removed, 128 of the 206 identified records were excluded after screening the title and abstract. In addition, one record was excluded because it was neither open access nor accessible via the University of Geneva/Swisscovery Network. Of the 54 full-text articles assessed for eligibility, 18 were excluded because they did not meet the inclusion criteria (Figure 1). No articles written in French that responded to the selection criteria were identified.

A total of 36 articles were included in our review: nine randomized control trials (RCTs), five cohort studies, 19 qualitative studies, and three socioeconomic studies, all associated with an RCT to specifically assess the cost-effectiveness of an SMS intervention versus standard of care (Supplementary File S3).

Of the 36 included articles, 32 articles included adults aged 18 and over, two included young people aged 15–24 years, one included young people aged 12–19 years, and one included people aged 16 and over.

In addition, the HIV epidemic profile and related country response and the level of digitization of health were found to be important factors influencing the selection of the mHealth solution, study design, and health outcomes. This is particularly true for qualitative studies.

### Geographical Scope

Only two studies conducted in West and Central Africa (WCA) were selected as compared to 34 in East and Southern Africa (ESA), confirming that mHealth-related research and project implementation in francophone countries in WCA still lags behind [15]. However, the results obtained in these countries are similar to those obtained in anglophone Africa [16]. Three countries (South Africa *n* = 15; Uganda *n* = 6; and Kenya *n* = 6) account for 75% (27/36) of the total number of selected studies, confirming their monopoly on research in this field.

### Study Outcomes on Effectiveness

Six (16.7%) studies focusing on testing uptake were identified: two (5.5%) on linkage to care, two (5.5%) on retention in care and/or adherence to treatment, and six (16.7%) on viral load monitoring or laboratory results transmission.

Fourteen studies confirmed the growing evidence of the benefits of mHealth in increasing HIV testing uptake, linkage to care after HIV self-testing or HIV home-based testing, and transmitting viral load results via SMS, measured by retention in care or viral suppression after several months. No negative outcomes were reported in the intervention areas. Two studies with similar outcomes that showed limited or no impact on adherence to ART and retention in care were identified [17, 18]. However, the authors of these two studies measured additional benefits using qualitative indicators and found an increase in self-perceived well-being. Three studies did not assess the specific impact of SMS messaging as it was included in a broader strategy. They demonstrated the effectiveness of a combinatorial intervention strategy.

### Patient-Reported Outcomes on SMS-Based Interventions

Among the 36 selected studies, 32 were designed for basic or feature cell phones and 20 used an automated SMS service, mainly to send appointment reminders and/or non-personalized disease prevention and health promotion information. Even when a difference was not shown between the SMS intervention and control arms in terms of retention in care or adherence to treatment, beneficiaries receiving SMS messages felt better taken care of by their healthcare provider. This has resulted in improved interaction between patients and healthcare providers and an increase in patients’ perceived well-being [16, 18–21]. Participants living with HIV in the only RCT using simple feature phones and two-way communication with interactive voice response technology using a pre-recorded voice suggested that they became attached to the “voice” and felt as if they were better cared for [17].

### Technology Choice for the mHealth Solution

Two studies used the Wisepill antiretroviral (ARV) dispenser with global system for mobile communication (GSM) [22, 23]. One study used mobile cash transfer [24], and another study assessed the acceptability and feasibility of using an iPhone among female sex workers [25]. The authors showed that even if the fingerprint scanning function of the iPhone could be an added value to increase privacy and security, the cost of such technology and access to the internet were identified as major barriers. Four studies had developed a specific web-based application which needed to be downloaded on a smartphone [26–29]. It was noted that the use of such technologies may compromise the results. For instance, in South Africa, Venter et al. reported a 20% increase in linkage to care for youth aged 18–30 years using a specifically developed app, but only after excluding 90% of interested young people living with HIV because they did not own an Android smartphone with the required specifications [30]. By contrast, DiAndreth et al. [31] excluded only 14% of people referred to the laboratory for viral load, either for not owning a basic cell phone with a 2G or 3G connection or for not being willing to be enrolled.

### Financial Acceptability for End Users

Communication costs can be a barrier to the use of mHealth by people living with HIV in some contexts. Studies offering incentives and/or airtime to participants to mitigate this barrier showed that provision of airtime cards was preferred over cash incentives [32]. Interventions associated with SMS messages with financial incentives showed no statistically significant difference between the SMS and SMS with incentive groups in the proportion registered at the clinic, initiating ART, or achieving viral suppression by study group [24, 32–34]. For the youth population, the incentive amount and type of incentive seemed to have a relatively lower importance than other attributes [35].

By contrast, PLHIV in Côte d’Ivoire, Togo, and Burkina Faso were likely to financially support out-of-pocket expenditures generated by the adoption of an mHealth solution. No significant relationship was observed between financial acceptability and monthly income [16].

Finally, all three socioeconomic studies showed a cost-effective impact of the SMS interventions on the continuum of care. Such interventions were considered by the investigators a good value for the money [36] and valuable in contexts similar to that of the study [37]. Interventions are more cost-effective in the context of a multicomponent strategy, as the achieved impact is greater [38].

### Perspectives From Grey Literature on Projects Currently Implemented in SSA

The scans of the WHO Digital Health Atlas, the GSMA mHealth Tracker and the Pierre Fabre Foundation’s eHealth Observatory identified a total of 21, 138, and 17 HIV/AIDS earmarked mHealth projects currently being implemented in SSA, respectively. Out of the 175 projects identified, after duplicates were removed, 23 projects implemented in six countries were selected.

It is interesting to highlight that 138 (78.8%) of the identified projects, and 17 (73.9%) of the selected projects, have been implemented in partnership with one or more mobile network operators, a technology firm, start-up, or pharmaceutical company through a public–private partnership, or a public–private–civil society partnership. Five projects directly implemented by mobile network operators were identified. However, the narratives and data provided on the three websites are insufficient to understand the scale, level of deployment, and impact, of different projects, or establishment of sufficiently informative mapping. Furthermore, information on whether the mHealth solution is a proprietary license or a protective free and open-source software is often not provided. Thus, these databases merely provide an overall picture of the importance of digitizing the HIV response in SSA.

### Toolkits and Guidelines From Grey Literature

The WHO has published several guidelines and handbooks for general orientation or related to the management of a specific disease, such as tuberculosis (TB), cervical cancer, and tobacco cessation. No material dedicated solely to the AIDS response could be identified. The same conclusion applies to John Hopkins University Global mHealth Initiative, Digital Square, and GSMA repositories.

## Discussion

### Primary Objective

Our scoping review confirms the increased digitalization of health and the growing evidence of the benefits of using mobile phones to improve the continuum of care by linking PLHIV to care to initiate ART after undergoing an HIV test, regardless of the HIV testing method applied and retaining them in treatment until they present with a suppressed viral load. The review also demonstrates the important cross-cutting benefits of mHealth in fostering a renewed patient-healthcare provider interface, thus leading to improved patient empowerment.

In parallel, the review revealed a gap between research and implementation, particularly in collaboration with mobile network operators and other private sector representatives, as well as gaps in normative guidance.

First, the review suggested the persistence of an mHealth francophone gap and the dominance of a few ESA countries [15]. This can be partially explained by the ICT infrastructure and ecosystem, with the early adoption of optical fiber through submarine cables in East Africa and the Republic of South Africa; massive investments in digital health projects from the United States Agency for International Development [39], political commitment by governments that are early promoters and adopters of digital health; and the presence of robust universities and research institutions. In parallel, the level of income and associated political willingness to achieve the Abuja target of 15% domestic expenditure on health [40] and the resilience of the health system remain important factors. The dual challenge of equity in access to health services and mobile phone services constitutes a complex and ever-changing ecosystem that can act as a break for the deployment and adoption of mHealth solutions. It is important to consider how mHealth can contribute to reducing inequities in access to health services, not only between anglophone and francophone countries, but most importantly within a country, between urban and rural settings, by linking individuals to the health system. At the same time, it is necessary to ensure that mHealth will not create additional inequities based on factors such as connectivity, network coverage, access to mobile internet, and mobile phone possession.

**FIGURE 3:**
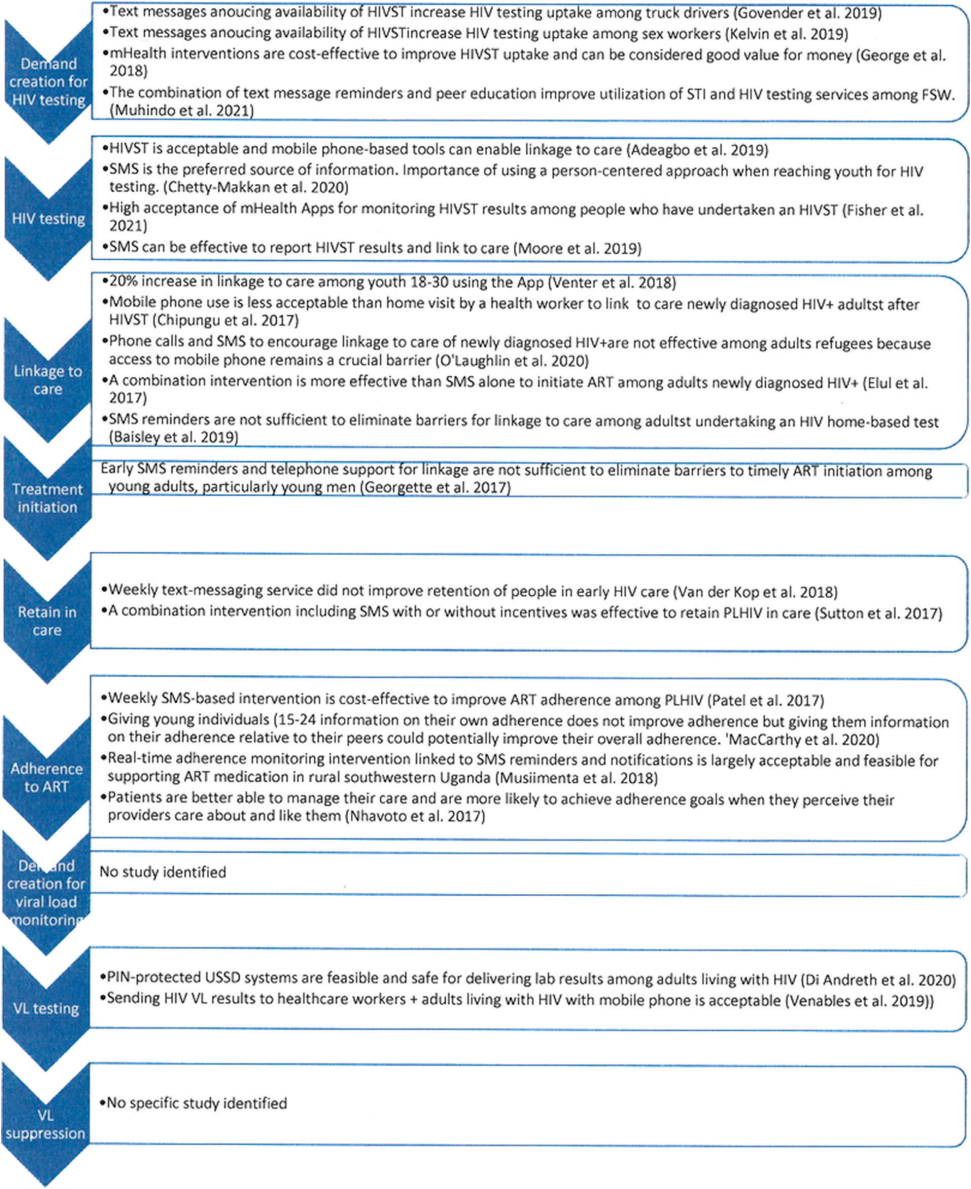
Major lessons learnt regarding the HIV continuum of care from identified studies (scoping review, Sub-Saharan Africa, 2017–2021).

**FIGURE 4:**
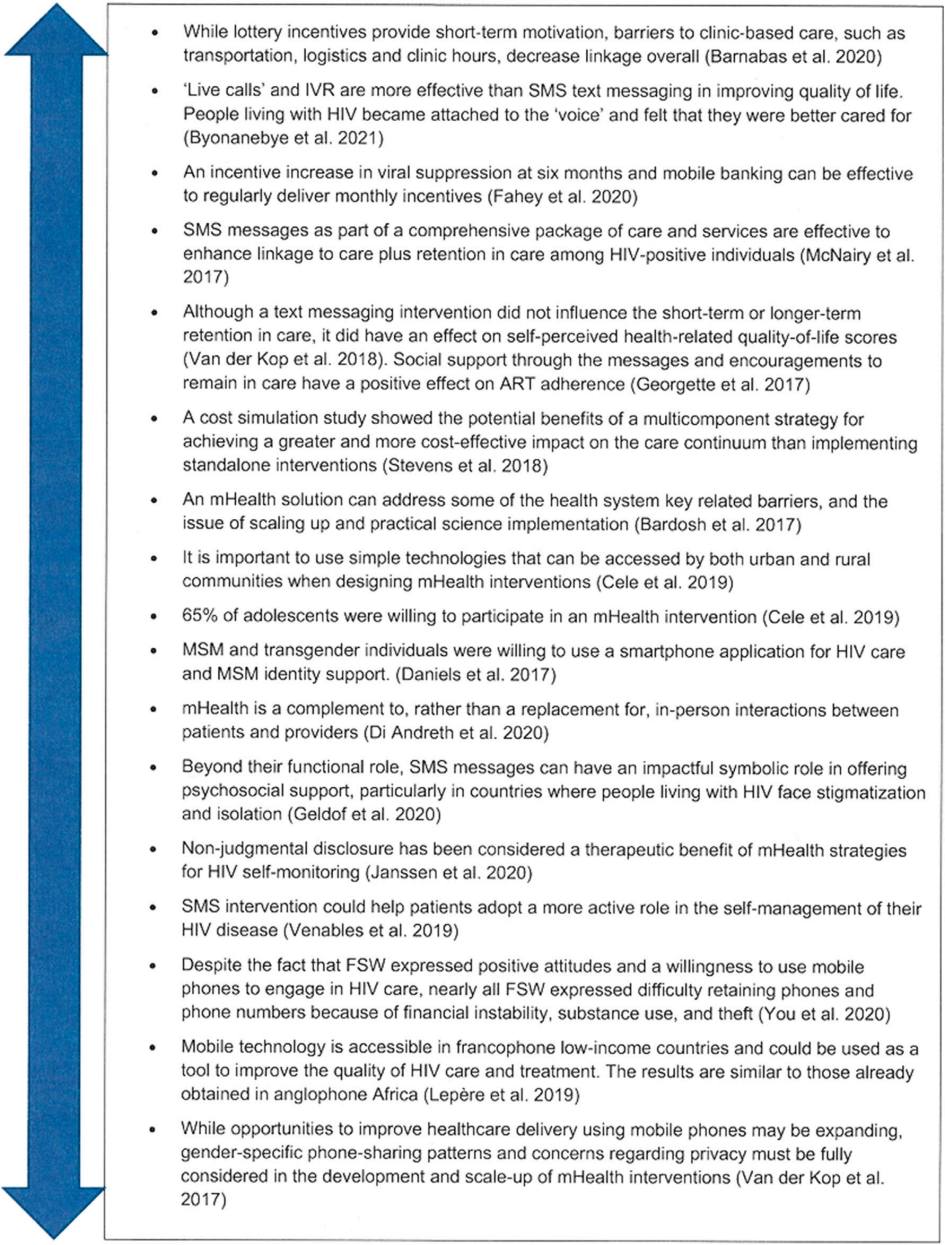
Major lessons learnt regarding cross cutting issues from identified studies (scoping review, Sub-Saharan Africa, 2017–2021).

Second, we found a high number of studies conducted in silos (Figure 3) and a few identified studies evidenced findings that should be considered at different stages of the continuum of care (Figure 4). No research interventions proposing to experiment with different mHealth tools simultaneously to address each of the three 90/95 targets in a holistic manner were identified. Considering the WHO recommendations for research to examine the synergies across different combinations of digital health interventions [14, 41], the review suggests breaking the silos and better exploring the potential of mHealth by proposing more holistic approaches to the HIV treatment cascade and expanding the scope of selected mHealth solutions. Interestingly, few studies have used mobile money tools in the field of health as an appropriate solution to deliver incentives.

Finally, while the WHO recommends research to explore the cost-effectiveness, potential for cost-saving interventions, and additional savings allowed by implementing a combination of interventions [41], this review found a gap in planning the necessary financial and economic studies.

### Secondary Objectives

Application of principles for digital development (Figure 2) allowed us to refer to a common framework and identify key lessons learned to translate science into strategies or policies.

In 2014, a systematic review aimed at identifying factors influencing the success and failure of mHealth projects in Africa and concluded that an effective public, private, and civil society partnership was among the keys to success [42]. Moreover, in 2016, the WHO clearly recommended exploring public–private partnerships to accelerate the scaling-up of mobile phone-based interventions [5]. Despite these recommendations, research studies rarely involve mobile network operators, nor are they conducted through a public–private partnership. In addition, many studies interventions that developed their own digital solutions instead of opting for a protective free and open-source solution were identified. Understanding the local mHealth ecosystem remains essential before conducting new research, and collaboration with mobile network operators can ease this process. Research teams could learn how to design studies and digital solutions from development partners and implementers in collaboration with mobile network operators. In the absence of such learning, there is a risk of hiatus between science and implementation.

Second, in the scheme of the joint vision of the WHO-United Nations International Children’s Emergency Fund for primary health care in the 21st century [43] and the WHO’s five-strategy framework on integrated, people-centered health services [44], the scoping reviews suggests that bulk SMS messages may not constitute the most appropriate tool for patient empowerment, as these do not allow bi-directional communication, sufficiently reinforce the patient/clinician interface or fully respond to the end user’s need to develop a personalized relationship [21, 45, 46]. Future research studies should consider bi-directional communication to improve the clinician/patient interface, health literacy and digital health literacy, and supply-side users’ competencies, and respond to health-care providers’ quest for better working conditions. Co-construction that connects supply-side users and end-users in the design of such studies is a *sine qua non* for positive and sustainable outcomes.

### mHealth in the Era of the COVID-19 Pandemic

Finally, this scoping review was conducted during the coronavirus disease pandemic. Therefore, it is important to consider the consequences of this pandemic on both the AIDS response and mHealth. mHealth has been widely used in SSA in the context of the COVID-19 crisis to communicate messages about preventive measures [47, 48]. mHealth tools have been proven to add value in terms of informing patients about test results without using mobile data. Healthcare providers working in HIV and TB programs who were used to communicating with their patients via mHealth were comfortable managing severe acute respiratory syndrome coronavirus two infected individuals’ self-isolation remotely. For example, the WelTel platform, an evidence-based text messaging solution initially designed to improve HIV treatment adherence in Kenya, is currently being deployed in response to COVID-19 in Rwanda, Uganda, Tanzania, and the UK for contact tracing and the home-based care of positive cases [18, 49, 50]. It appears that lessons learned regarding the use of mHealth in the framework of the HIV response have benefited the COVID-19 response. Lessons learned regarding mHealth use during the current COVID-19 pandemic should allow the HIV response to make a leap forward. Despite the many disruptions in health systems resulting from COVID-19 worldwide, the pandemic should offer an opportunity to accelerate the development of mHealth tools in LMICs and reinforce the management of chronic diseases.

### Implications for Practice and Policy

A conducive environment that includes digital health solutions in a package of care is essential to enable the use of mobile phones in public health programs. Relevant approaches include patient empowerment and ease of access to services through a rebuilt and trusted patient/healthcare provider relationship. Research studies should be co-created with the involvement of both end-users and healthcare providers to respond to identified needs or bottlenecks in the continuum of care. Economic and financial analyses should be included in the early stages of the study protocol.

The scoping review showed that research studies rarely mention the level of compliance of mHealth interventions to the national digital health strategy, policy, or framework in the relevant location. In the absence of such a strategy, policy, or framework, research findings should underline the lessons learned to inform the development of such an important national document. LMICs need scalable programs that can be integrated into their national health systems, compliant with their national eHealth policies and international guidance, and preferably designed and implemented through a public–private–civil society partnership with mobile network operators.

### Strengths and Limitations

To the best of our knowledge, this original scoping review was conducted with a holistic approach to the HIV treatment cascade, focusing on the patient/healthcare worker interface, and is positioned at the crossroads between the key turning point in the AIDS response in 2016 and the promotion of the use of mHealth solutions by the WHO.

The review shows an evolution of research topics in line with the concomitant evolution of both the digital health field and the HIV response. By orienting the discussion towards scientific implementation, the findings provide the necessary evidence to inform decision-makers and implementers. In addition, this review calls on researchers to consider this perspective in future studies.

One limitation of this study is that the initial selection of the included documents was not carried out by multiple researchers to ensure to reflect the entire body of research. While Arksey and O’Malley did not recommend that more than one researcher conduct study selection [9], Levac et al. encouraged the use of a team approach [51]. We believe that a team approach was an essential component of this review, as the co-authors supported the first author regarding the rationale and relevance of the screened studies. Nonetheless, we believe that a systematic and rigorous search strategy was applied to retrieve relevant articles responding to the research question, as all data extracted were shared for verification by all other authors.

### Conclusion

Our study revealed that mobile phones can contribute to improving the relationship between healthcare providers and patients for better health outcomes and greater satisfaction of both the patient and healthcare provider. Owing to mHealth, patients are more likely to adopt an active role in the self-management of their HIV infection. In parallel, a gap between research and implementation was identified, particularly in collaboration with mobile network operators and other private sector representatives, as well as in normative guidance.

Finally, this scoping review confirms the onwards march of the digitalization of health and the growing evidence of the benefits of using mobile phones to improve the continuum of care. mHealth should substantially contribute to the achievement of the 95-95-95 targets in SSA by 2025 if the local complex and ever-changing ecosystem, including the socioeconomic dimension, were better considered to address the dual challenge of equity in access to health services and mobile phone services.

## Footnotes

1
https://www.unaids.org/en/aboutunaids/unitednationsdeclarationsandgoals/2016highlevelmeetingonaids.

2
https://digitalprinciples.org/principles/.

3
https://digitalhealthatlas.org/en/-/dashboard/?country=157.

4
https://www.gsma.com/mobilefordevelopment/m4d-tracker/mhealth-deployment-tracker/.

5
https://www.odess.io/the-observatory.html.

6
https://www.who.int/observatories/global-observatory-for-ehealth.

7
https://www.who.int/health-topics/digital-health#tab=tab_1.

8
https://lib.digitalsquare.io/handle/123456789/6.

9
https://www.jhumhealth.org.

## Abbreviations

AIDS, acquired immunodeficiency syndrome; ART, antiretroviral therapy; EID, early infant diagnosis; ESA, East and Southern Africa; GSMA, Global System for Mobile Communications Association; HIV, human immunodeficiency virus; ICT, information and communications technologies; LMICs, low- and middle-income countries; PMTCT, prevention of mother-to-child HIV transmission; PRISMA, Preferred Reporting Items for Systematic Reviews and Meta-Analyses; RCT, randomized control trials; SMS, short messaging service; SSA, sub-Saharan Africa; UNICEF, United Nations International Children’s Emergency Fund; WHO, World Health Organization; WCA, West and Central Africa.
