# Supplementary material for: How mHealth Can Contribute to Improving the Continuum of Care: A Scoping Review Approach to the Case of Human Immunodeficiency Virus in Sub-Saharan Africa
Source: Public Health Rev. 2022 Sep 23;43:1604557. doi: 10.3389/phrs.2022.1604557 (PMC9537374; doi:10.3389/phrs.2022.1604557)
Supplement: Supplementary file 1 [file DataSheet4.docx]

**Supplementary file 4: Main characteristics of identified projects (scoping review, Sub-Saharan Africa, 2017 – 2021).**

| **Name** | **Location** | **Implementing organization** |  | **tool properties** |  | **Partnerships** |  | **Goal of the intervention** | **Nature of interventions** | **Results** |
| --- | --- | --- | --- | --- | --- | --- | --- | --- | --- | --- |
| Connect for life | Uganda | Infectious Disease Institute (Academia) |  | IVR software with proprietary license |  | PPP with Johnson & Johnson (pharmaceutical company) |  | To improve virologic outcomes positively impacting quality of life. | Daily pill reminder and health tips and support management of medication side-effects along with HIV knowledge | No data |
| Improving outcomes in HIV patients using mobile phone based interactive software support | Uganda | Infectious Disease Institute (Academia) |  | Protective free and open source software |  | PPP with Johnson & Johnson (pharmaceutical company) |  | To support adherence to HIV treatment | Clinic appointment reminders, self-reported adherence and symptom reporting. | No data |
| Sharper | Ghana | FHI 360 (NGO) |  | SMS platform with proprietary license |  | with Ghana AIDS Commission (Gov) |  | To provide HIV prevention, treatment and care services | ART reminder and counselling | No data |
| Employee quizzes for HIV/AIDS | Kenya | Airtel Kenya (MNO) |  | No data |  | Project directly implemented by the MNO |  | To encourage HIV testing uptake | Motivational text messages (SMS) and quizzes (USSD) | No data |
|  | Uganda | Airtel Uganda (MNO) |  | No data |  | Project directly implemented by the MNO with MSD/Merck & Co, Inc (Pharmaceutical company) |  | To encourage HIV testing uptake | Motivational text messages (SMS) and quizzes (USSD) | No data |
| Grand challenges Canada phase II | Kenya | AMREF (NGO) |  | No data |  | With Weltel Kenya (private sector) and universities (academia) |  | To scale-up findings from an RCT aiming at supporting medication adherence | Weekly short SMS text-messages | No data |
| HIV/AIDS Program | Kenya | Airtel Kenya (MNO) |  | No data |  | Project directly implemented by the MNO |  | To encourage HIV testing uptake and provide treatment and care services | SMS text messaging | No data |
|  | Uganda | Airtel Uganda (MNO) |  | No data |  | Project directly implemented by the MNO with PharmAccess Foundation, Uganda (PPP) |  | To encourage HIV testing uptake and provide treatment and care services | SMS text messaging | No data |
|  | Zambia | Airtel, Zambia (MNO) |  | No data |  | Project directly implemented by the MNO with PharmAccess Foundation, Zambia (PPP) |  | To encourage HIV testing uptake and provide treatment and care services | SMS text messaging | No data |
| Increasing uptake of HIV treatment using SMS | Uganda | Text to change, Netherlands (NGO) |  | No data |  | with Dutch Embassy Uganda (Donor) |  | To increase uptake of HIV treatment | Text-messaging service | No data |
| Measurement of adherence to ART | Uganda | Mbarara University of Science and Technology (Academia) |  | SMS and IVR with Wisepill dispenser |  | with Massachusetts General Hospital, USA (Academia) |  | To monitor treatment adherence | Text-messaging and IVR services | No data |
| Results SMS | Uganda | Support for International Change (NGO) + Global Partners in Anesthesia and Surgery (Academia) |  | Open-source platform for SMS-based results |  | with ResultSMS, India (Private technology firm) |  | To deliver lab result with SMS | Text-messaging service | No data |
|  | Uganda | Appfrica (Private technology firm) |  | FrontlineSMS platform (paid-service) for SMS-based results |  | with Harvard University, Uganda (Academia) |  | To deliver lab result with SMS | Text-messaging service | No data |
| SMS adherence programme | Uganda | Kawempe Home Care (NGO) |  | No data |  | with Text to change, Netherlands (NGO) and USAID (Donor) |  | To support medication adherence | Text-messaging service | No data |
| SMS to improve HIV treatment adherence | Uganda | Text to change, Netherlands (NGO) |  | No data |  | with MSD/Merck & Co, Inc (Pharmaceutical company) |  | To provide HIV prevention, treatment and care services | Text-messaging service | No data |
| Kgakololo Project | Botswana | Positive innovation for the Next Generation, (Association of PLHIV) |  | No data |  | PPP with the Ministry of Health and the MNO Mascom |  | To support medication adherence | Text-messaging service | No data |
| Adherence messaging | South Africa | Cell-Life (University of Cape Town) (Academia) |  | No data |  | with University of Durban (Academia) |  | To support medication adherence | Text-messaging service | No data |
| Development and implementation project | South Africa | Desmond Tutu HIV Foundation (DTHF) |  | iDART software (Protective free and open-access solution) |  | with Universities of Durban, Cape Town and Cape Peninsula (Academia) |  | To support ARV dispensation | No data | No data |
| Project Masiluleke | South Africa | Praekelt Foundation (NGO) |  | SMS and IVR |  | PPP with the MNO MTN and the Nokia Siemens Networks firm |  | To provide HIV prevention, treatment and care services | Text-messaging service | No data |
| Txt alert | South Africa | Praekelt Foundation (NGO) |  | SMS + helpline + call-back system |  | PPP with MTN (MNO) and the National AIDS Helpline (Gov) |  | To support medication adherence | Automated, personalized SMS reminders | No data |
| Txt alert at Helen Joseph hospital | South Africa | Praekelt Foundation (NGO) |  | SMS + helpline + call-back system |  | PPP with MTN (MNO) and the National AIDS Helpline (Gov) |  | To support medication adherence | Automated, personalized SMS reminders | No data |
| Txt alert: ARV for Essellen clinics | South Africa | Praekelt Foundation (NGO) |  | SMS + helpline + call-back system |  | PPP with MTN (MNO) and the National AIDS Helpline (Gov) |  | To support medication adherence | Automated, personalized SMS reminders | No data |
| Txt alert: ARV for Malvern clinics | South Africa | Praekelt Foundation (NGO) |  | SMS + helpline + call-back system |  | PPP with MTN (MNO) and the National AIDS Helpline (Gov) |  | To support ART uptake after HIV testing | Automated, personalized SMS reminders | No data |
