# Supplementary material for: How mHealth Can Contribute to Improving the Continuum of Care: A Scoping Review Approach to the Case of Human Immunodeficiency Virus in Sub-Saharan Africa
Source: Public Health Rev. 2022 Sep 23;43:1604557. doi: 10.3389/phrs.2022.1604557 (PMC9537374; doi:10.3389/phrs.2022.1604557)
Supplement: Supplementary file 2 [file DataSheet3.docx]

**Supplementary file 3: Main characteristics of identified studies (scoping review, Sub-Saharan Africa, 2017 – 2021).**

**Qualitative studies**

| **First author and year** | **Journal** | **Location** | **Study design** | **Sample characteristics** | **Tool properties** | **Goal of the intervention** | **Outcomes** | **Results** |
| --- | --- | --- | --- | --- | --- | --- | --- | --- |
| Adeagbo et al. 2019 | JMIR | South Africa | Candidacy framework. In-depth interviews and focus group discussions | 54 Community members +  9 Healthcare providers (Both sexes aged 18-79) | App for individuals to identify and test their candidacy for HIV services. | To codevelop (with potential users) a new generation of mobile phone–connected HIV diagnostic tests and Web-based clinical care pathways needed for optimal delivery of decentralized HIV testing, prevention, and care in low and middle-income countries. | Participants’ willingness to use an mHealth technology for HIV testing and treatment | HIVST is acceptable and mobile phone-based tools can enable linkage to care. |
| Bardosh et al. 2017 | Globalization and health | Canada and Kenya | In-depth interviews with stakeholders involved in an ongoing WelTel project | 32 Stakeholders involved in Weltel projects | Tool = Weltel internet-based platform. Automated weekly non-personalized SMS sent and follow-up via phone calls when necessary. Two-ways communication | To explore how the WelTel intervention was perceived, diffused, adopted and used by different health system actors in Canada and Kenya. | Influence of the intervention on the patient-provider relationship. | Greater access to care through reinforced relationships between user and provider. Bidirectional communication improves the culture of care at clinic level and clinician accountability |
| Cele et al. 2019 | Southern African Journal of HIV Medicine | South Africa | Cross-sectional observational study in a rural and urban clinic | 100 adolescents (12 – 19 years old) living with HIV on ART – median age 15 | No tool at this stage | To assess specificities related to adolescents before designing an intervention and compare rural and urban settings | Acceptability of text messaging support for adolescents in an urban and rural clinic | 65% of adolescents were willing to participate in a mHealth intervention. |
| Chipungu et al. 2017 | Plos One | Zambia | Cross-sectional survey + focus group discussions | 1617 individuals aged 16-49 years old – median age 27 randomizely selected from population housing and census | Not accurate | To define the preferred linkage strategy after self-testing. | Acceptability of mobile phone use and intention to link to care of individuals who got diagnosed HIV + after undertaking an HIVST | Overall, 53% (95%CI = 50 to 55) of respondents reported preferring a home visit from a health care worker, and 30% preferred a phone call, followed by 17% who preferred SMS |
| Daniels et al. 2017 | JIAPAC | South Africa | Questionnaire + focus group discussions | 18 community leaders representing MSM and transgender individuals | Not accurate | To assess specificities related to MSM and transgender individuals in the "how" they use their mobile phone and interest in participating in a research work | Feasibility of using mobile phone and SMS for HIV care research interventions with this community | Most participants were willing to use SMS functions including receiving messages and responding to these within a research study (83% for all), and all participants were willing to use a smartphone application for HIV care and MSM identity support. Monthly in-person HIV support groups, with SMS reminders to attend these groups, were of interest to 94% of the participants. |
| Di Andreth et al. 2020 | AIDS behav | South Africa | Unmasked non-randomized study | 444 referred adults (aged ≥ 18 years – median age 37 years old) who had ≥ 1 laboratory sample taken for a CD4 count, VL assay, or TB Xpert MTB/RIF (Cepheid), owning a mobile phone or having access to, and literate | MatlaMobile is compatible with both smart and basic phones. Dedicated Microsoft web-based technology to deliver CD4, VL, and TB results to patients’ mobile phones. MatlaMobile used an unstructured supplementary service data (USSD) system for participants to access PIN-protected messages that, unlike SMS, were not stored locally on the mobile phone but could still be viewed repeatedly. MatlaMobile also offered options for participants to request a call-me-back from a nurse or send their laboratory result to themselves via SMS text message. | Intervention: SMS notifications to inform the availability of lab results and possibility to check them via USSD + daily SMS reminders | Participants using their mobile phone to see lab results and returned to clinic vs returned to clinic in the control arm. | Participants viewed their test result within 7 days of their enrollment (73.0%) compared to the control arm (8.6%, p < 0.001). In this study, an mHealth program using USSD successfully and securely delivered CD4, VL, and TB laboratory results to patients’ mobile phones in South Africa. |
| Di Andreth et al. 2020 | AIDS Care | South Africa | Qualitative in-depth interviews | 28 providers (nurses and doctors) and 11 adults living with HIV attending an HIV clinic | Not accurate | To identify perceived benefits, concerns and recommendations from patients and providers for the design and implementation of an mHealth program to deliver HIV viral load and CD4 count test results directly to patients via mobile phone. | Acceptability of sending HIV test results directly to patients through their mobile phone | reduced workload for providers and reduced wait time for patients. Providers worried patients may feel stress when receiving results without direct support from providers, and patients were wary of losing in-person interaction with healthcare providers. Both patients and providers felt that patients should have a choice over how to receive results (via mobile phone or in-person). |
| Fischer et al. 2021 | Southern African Journal of HIV Medicine | South Africa | Cross sectional study + telephone post-study survey | 751 adults from general population who have undertaken an HIV self-test at home | Mobile-phone web-based application specifically designed to self-report an HIVST result | Feasibility studies have shown high acceptance of mHealth apps for the monitoring and evaluation of HIVSTs, but the reporting of results needs to be assessed | Self-reporting of HIVST result with the Ithaka app | 295 (39.3%) enrolled participants completed the pre- test counselling and the how-to-test instructions, and 168 (22.4%) enrolled participants self-reported their results. |
| Geldof et al. 2020 | AIDS Care | Burkina-Faso | Qualitative study (through semi-structured interviews + focus group discussions) | 75 HIV positive individuals aged >15 receiving an ARV therapy enrolled in a RCT employing SMS messages to increase retention and adherence | Not accurate | Qualitative exploration of the mechanisms underlying the intervention + exploration of potential for the symbolic role of SMS messages as means to provide psychosocial support to PLHIV | Qualitative assessment of the impact of SMS messages | Beyond their functional role, SMS messages can have an impactful symbolic role in offering psychosocial support, particularly in countries where PLHIV face stigmatization and isolation. |
| Janssen et al. 2020 | AIDS Behavior | South Africa | Qualitative study through 30 semi-structured interviews + one focus group discussion with study staff at primary health facilities | 16 adult females and 14 adult males willing to undertake a HIVST | Having a smartphone (Android or IOS) and downloading the dedicated App + assistance of study counselors | To examine the interaction between a smartphone based HIVST app, in a supervised and unsupervised HIVST context, and HIV self-testers in South Africa. | Characterization of participant's experiences and practices using an App to help them familiarizing with HIVST testing technic | The App helps people getting pre-test counselling, and care and simplifying the process of self-testing. The App allows to overcome barriers related to lack of confidentiality, waiting time at clinic. Privacy at home remains a concern. |
| MacCarthy et al. 2020 | BMC Infectious diseases | Uganda | Focus group discussions using behavioural economics for qualitative analysis and preliminary data of the RCT for quantitative analysis | 155 young PLHIV aged 15 - 24 receiving ART and enrolled in a RCT. Patients demonstrating less than 20% adherence were excluded | Wisepill dispenser with GSM communication (if patients forget to take their medication at the prescribed time, a text message reminder is sent to their mobile phones). 13% of participants were excluded during the pre-baseline survey because they did not use the Wisepill device consistently. | To explore specificities related to youth 15 - 24 years by assessing their tendency to overestimate their capabilities and their tendency of equalling or surpassing the performance of their peers | Acceptability, feasibility, and preliminary impact of the intervention | Giving individuals information on their own adherence does not improve adherence but giving them information on their adherence relative to their peers could potentially improve their overall adherence. |
| Musiimenta et al. 2018 | JMIR mHealth and uHealth | Rural Uganda | Semi structured qualitative interviews | 63 adults PLHIV initiating ART median age 30 years and their social supporters (41) participating in a RCT based on real-time intervention linked to SMS reminders and notifications to support adherence among PLWHA taking ART | Dimagi SMS system hosted by the CommCare application + the Wisepill medication dispenser | To assess the acceptability and feasibility of real-time adherence monitoring linked to text messaging (short message service, SMS) reminders and notifications to support adherence among individuals living with HIV who are taking ART in rural southwestern Uganda | Acceptability and feasibility of the intervention | Real-time adherence monitoring intervention linked to SMS reminders and notifications is largely acceptable and feasible for supporting ART medication in rural southwestern Uganda |
| Nhavoto et al. 2017 | Plos One | Mozambique | Telephone interviews with patients and on-site interviews with health care workers | 72 adults living with HIV and 69 adults infected with TB enrolled in the intervention arm of a RCT with median age of 38 years + 40 HCWs | Automated SMS messages as appointment reminders + two-ways communication allowing patients to ask question and receiving an answer from HCW | This study investigates perspectives of patients and HCWs regarding SMS use in a randomized control trial (RCT) aiming at improving patient retention in HIV and TB-HIV care | Patients' and health-care workers' perceptions towards the SMS system | SMS messages may encourage better quality of care for PLHIV and TB patients and may indeed be a useful addition to existing services |
| Venables et al. 2019 | Plos One | Zimbabwe | Qualitative study (in-depth interviews + focus group discussions) | People living with HIV aged 18 to 60, receiving ART and having participated in the SMS intervention group + their health care workers at facility level in 2 districts of Harare. 43 patients included in focus group discussions | Automated SMS message informing participants about the availability of VL results at the clinic. Patients were never sent messages with their actual VL results | To explore patient and health-care worker experiences of the intervention that consisted in transmitting the VL lab results via SMS | Acceptability and impact of the intervention | Median time from reporting of the VL result at the laboratory, to start enhanced adherence counselling reduced from 47 days (IQR 29–71) to 30 days (15.3–51.5) after the introduction of the intervention. SMS is an acceptable form of communication for patients to receive information about the availability of their VL results. |
| You et al. 2020 | Mhealth | South Africa | Qualitative study using semi-structured interviews through focus group discussions | 22 cisgender adults (> 18 years) FSW newly diagnosed HIV positive and ART naïve enrolled in the Syyaphambili trial and selling sex as the main source of income | Use of an Apple iPhone with more advanced digital technologies, including biometrics as tools for identification, mobile phone apps, and chatbots. | To identify potential facilitators and barriers faced by cisgender FSW living with HIV in South Africa in using mobile phone and other advanced digital interventions for HIV care. | Perceived usefulness, attitudes towards and control over digital health technologies for engagement in HIV treatment support | Despite that FSW expressed positive attitudes and a willingness to use mobile phones to engage in HIV care, nearly all FSW expressed difficulty retaining phones and phone numbers as a result of financial instability, substance use, and theft. |
| Chetty-Makkan et al. 2020 | AIDS and Behavior | South Africa | Discrete Choice Experiment (DCE) study | 130 young people aged 15-24 enrolled in the DREAMS project that aimed at reducing new HIV infections among young people | NA | To quantify HIV Testing Services (HTS) preferences among youth to inform the design of HTS services for better uptake and acceptability. | Participants’ choice | SMS is the preferred source of information. Importance of using a person-centered approach when reaching youth for HIV testing. |
| Muhindo et al. 2021 | BMC health services research | Uganda | Nonrandomized quasi-experimental study with pre- and post-test measurement surveys | 236 female sex workers who are not aware of their HIV status and not on ART, median age 26 years | Automated SMS | To assess the effect of peer education and text message reminders on uptake of regular syphilis and HIV screening services among FSW in Uganda | Effect of the intervention on uptake of regular syphilis and HIV among FSW | The likelihood of testing in the prior three months was lower among FSW who did not receive a text message reminder or peer education (28 % vs.17 %) when compared with FSW who received a text reminder or peer education [aPR] 0.72, 95 % CI: 0.51–1.02 and [aPR] 0.83, 95 % CI: 0.58– 1.19) respectively |
| Lepère et al. 2019 | JMIR mHealth and uHealth | Burkina-Faso, Côte d’Ivoire and Togo | Multicounty cross-sectional study | 1131 people living with HIV aged ≥18 years, median age 44, receiving ART for at least 1 year, and receiving follow-up at one of the 6 HIV clinics participating in the study | Not addressed | To explore acceptability and feasibility of an mHealth intervention for the improvement of care among PLHIV in Francophone West Africa countries. | Social and financial acceptability of an mHealth intervention to improve the quality of HIV care | 98.8% acceptability with no variations by country A total of 386 (34.9%), 708 (63.9%), and 11 (1.0%) patients were willing to receive phone calls only, text messages and phone calls, and text messages only, respectively. 50.1% (n=322), 88.7% (n=212), and 92.8% (n=231) of PLHIV in Côte d’Ivoire, Togo, and Burkina Faso, respectively, likely to financially support out-of-pocket expenditures generated by the adoption of a mHealth solution, if any. |
| Van der kop et al. 2017 | Journal of Telemedicine and Telecare | Kenya | Cross-sectional study using baseline data collected from an RCT (WelTel Retain) | 700 adults aged > 18 tested HIV-positive and not receiving ART, living in large informal settlements in Nairobi | Automated SMS messages | To determine whether disparities in mobile phone access affected the ability to participate in an mHealth trial, and whether there were gender-based differences in shared phone use and participants’ concerns about text-messaging with their healthcare provider | Pre-trial analysis of data from the WelTel Retain study on retention in HIV care to assess gender-based differences in phone access, phone sharing and concerns about receiving text messages from a healthcare provider. | Men and women were equally likely to be excluded from participating in the trial because they did not meet the study’s phone-related criteria (39/378 (10.3%) of men and 71/690 (10.3%) of women; p-value = 0.989). Most participants did not have any concerns about receiving text messages from their healthcare provider. Only six participants (6/700; 0.9%), all women, responded that they had concerns |

**Randomized control trials**

| **First author and year** | **Journal** | **Location** | **Study design** | **Sample characteristics** | **Tool properties** | **Goal of the intervention** | **Outcomes** | **Results** |
| --- | --- | --- | --- | --- | --- | --- | --- | --- |
| Barnabas et al. 2020 | Journal of International AIDS Society | South Africa | Randomized prospective trial | 131 HIV positive men aged ≥ 18 r | two-ways motivational text-messages | To explore conditional lottery incentives to link men living with HIV to care, ART initiation and viral suppression over time | Linkage to the ART clinic, ART initiation and viral suppression at six months among the intention-to-treat population. | ART initiation = 76% in the text message group and 93% in the lottery plus SMS group. No statistically significant difference between the groups. Compared to motivational text messages alone, lottery incentives decreased the median time to ART initiation to 66 from 126 days. |
| Byonanebye et al. 2021 | JMIR | Uganda | open-label randomized controlled trial | 600 adults PLHIV | Simple feature phone and two-ways communication with IVR technology (pre-recorded voice) and a software that is based on open-source Mobile Technology for Community Health (MoTeCH) | To determine the acceptability and impact of an interactive voice response (IVR)–based patient support technology among people living with HIV in Uganda. | Difference in the change in the QoL at 12 months among ART-experienced people living with HIV in the intervention and control arms at the two study sites. | Statistical difference in the change in QoL at 12 months in participants enrolled in the intervention and control arms. Similarly, there was no difference in viral suppression rates in the two arms. However, there was an association between improved QoL as well as viral suppression and adherence to clinic appointments in participants who had moderate or high use of the tool. |
| Elul et al. 2017 | Plos Medicine | Mozambique | 2-arm cluster-randomized study | 2004 adults newly diagnosed with HIV in VCT clinics – median age 34 years | Automated SMS health messages and appointment reminders | To examine the effectiveness of a combination intervention strategy (CIS) composed of several scalable evidence-based interventions targeting the multiple and prevalent health system, structural and behavioral barriers that patients face across the HIV continuum. | Combined outcome of linkage to care within 1 month and retention at 12 months after diagnosis | 57% linked to care within 1 month of diagnosis and 37% retained 12 months after diagnosis at diagnostic facility. Most importantly, 89% linked at diagnostic facility the same day as HIV test in the intervention arm, vs 16% in the standard of care arm. 75% of participants incl. in the intervention arm were eligible for ART |
| Fahey et al. 2020 | The Lancet | Tanzania | 3-arm randomized control trial. | 530 HIV positive adults aged 18 years and over having initiated an ART within the past 30 days | Attendance monitoring and cash transfer delivery was done with the study’s tablet-based mHealth application, which linked biometric identification to an automated mobile payment system compatible with all mobile banking providers in Tanzania. Participants who did not have access to a mobile banking account received money in hand from a research assistant. | To evaluate the effect of financial incentives for clinic attendance on viral suppression in a low-income or middle-income country, using mobile health technology that linked biometric attendance monitoring to automated mobile payments upon monthly clinic visits. | Retention in care with viral suppression at 6 months after starting ART | 134 (73·0%) participants in the control group remained in care and had viral suppression at 6 months, compared with 143 (82·9%) in the smaller incentive group and 150 (86·1%) in the larger incentive group. No statistically significant difference between incentive arms. |
| Govender et al. 2019 | AIDS and Behavior | South Africa, Zimbabwe, and Mozambique | Cluster RCT Baseline survey + interview | 1783 long distance truck drivers and their assistants, and female sex workers aged 18 years and over | automated SMS messages. | To assess the efficacy of a SMS intervention in reducing HIV risk behaviours and increasing HIV testing rates among two key population: long distance truck drivers and sex workers. | Self-reported behaviour and testing uptake as a result of the SMS intervention | Respondents in the SMS arm had higher odds of having ever tested for HIV at follow up (95.6% vs. 88.6%; AOR 5.17, 95% CI 1.44–18.54, p = 0.01), were more likely to have tested for HIV in the previous 6 months (86.1% vs. 77.7%; AOR 1.72, 95% CI 1.11–2.66, p = 0.02) |
| Kelvin et al. 2019 | AIDS and Behavior | Kenya | RCT | 2196 female sex workers who are irregular HIV tester – median age 28.6 years old | automated SMS messages. However, text message was not very detailed and many receiving it may not have understood what it meant | To assess whether announcing the availability of HIV self-test kits in a clinic system in Kenya via text message would bring more female sex workers to the participating clinics for HIV testing compared to the standard of care text message reminder about HIV testing in general | HIV testing rate among sex workers who received SMS to inform them about availability of HIVST kits | Increased HIV testing rates by 1.9 in the 2 months following the initial text message However, the percent testing even with the self-testing intervention remained alarmingly low (10.8% in the intervention arm) |
| McNairy et al. 2017 | Plos Medicine | Swaziland | Cluster site-randomized trial | 2197 adults aged 18+ years newly tested HIV positive – median age 31 years old | Automated SMS appointment reminders sent from a central server. SMS reminders were sent 3 days prior to an appointment and after a missed appointment. | To examine the effectiveness of a combination intervention strategy (CIS) composed of several scalable evidence-based interventions targeting the multiple and prevalent health system, structural and behavioral barriers that patients face across the HIV continuum. | Combined outcome of linkage to HIV care within 1 month of HIV testing plus retention in care at 12 months from HIV testing among participants at the individual level. | The combined interventions were 50% more effective than the SOC in enhancing linkage to care plus retention in care among HIV-positive individuals. |
| Van der Kop et al. 2018 | The Lancet Public Health | Kenya | Unmasked randomized parallel group (WelTel Retain) Intention to treat analysis | 700 adults aged > 18 tested HIV-positive and not receiving ART, living in large informal settlements in Nairobi | Automated SMS messages + phone calls where necessary. | To examine whether weekly, interactive text messaging had an effect on patient retention during the first year of HIV care. | Effect of weekly, interactive text messaging on retention in care at 12 months versus standard of care | This weekly text-messaging service did not improve retention of people in early HIV care. The greatest perceived benefits were convenient access to care and advice (88 [40%] of 219); regular contact with health-care providers (54 [25%] of 219); and feelings of care, support, or security (42 [19%] of 218). |
| Venter et al. 2018 | JMIR mHealth and uHealth | South Africa | 2-arm multisite RCT | 181 men adults (18+) newly diagnosed HIV positive using the SmartLink App vs 164 adults receiving standards of care (SOC). | Smartlink App = Android-based platform. App not accessible on Google Play Store but can be downloaded only by confirmed HIV positive people. App installed by study staff. Participants received a 50 Rand (≈US $4) phone credit at recruitment to ensure all app-related costs were covered. | To test whether providing newly diagnosed HIV patients their laboratory results and supporting information securely on their mobile phones, via an app, would improve linkage to HIV care | Effectiveness of the application to improve linkage to care among youth aged 18 to 30 years | Youth aged 18 to 30 years, showed a statistically significant benefit of the app with a 20% increase in linkage to care for the app group. |

**Cohort studies**

| **First author and year** | **Journal** | **Location** | **Study design** | **Sample characteristics** | **Tool properties** | **Goal of the intervention** | **Outcomes** | **Results** |
| --- | --- | --- | --- | --- | --- | --- | --- | --- |
| O'Laughlin et al. 2020 | Global Public Health | Uganda | Cohort study | All adult refugees and Ugandan nationals aged > 18 participating in routine voluntary clinic-based HIV testing at Nakivale Health Centre. 101 individuals in the intervention cohort versus 107 individuals in the non-intervention cohort | phone calls and SMS for appointments reminders. | To evaluate a communication intervention for people newly diagnosed with HIV in a refugee settlement in Uganda to determine the feasibility and effectiveness of phone calls and text messages for clinic reminders in this unique population and setting. | HIV clinic attendance (i.e. ‘linkage’) within 90-days of HIV diagnosis | Phone call/SMS intervention to encourage linkage to HIV clinical care did not significantly improve linkage for all comers. Additionally, more than half (55%) of the participants in the Intervention group were illiterate and could not receive text messages. |
| Sutton et al. 2017 | J Acquir Immune Defic Syndr | Mozambique | 2-arm cluster site randomized study | People living with HIV undertaking a POC CD4 test | Automated SMS reminders for appointments sent to participants providing a phone number. SMS messages were sent to study participants using a Frontline database (Occam Technologies, Inc., Washington, DC) | To assess the feasibility and acceptability of health communication versus structural interventions as part of a combination intervention strategy. | Effectiveness of a combination intervention strategy (SMS reminder alone or SMS reminders + 3 months prepaid air-time cards) compared with the standard of care in improving linkage and retention among adults after HIV diagnosis. | Of 254 participants, retained in care at 12 months, 67% reported that the SMS reminders was the most useful intervention in facilitating retention and 24% reported that the financial incentive was the most useful intervention. Higher acceptability of the health communication intervention (SMS reminders) than of the structural intervention (financial incentives) |
| Moore et al. 2019 | Plos One | South Africa | Cohort study | 639 HIV negative individuals aged >18 years old willing to undertake an HIVST and report their results by pre-paid SMS | HIVST results reported by SMS were automatically captured in an electronic database. Results were instantly acknowledged by an automated SMS response. Participants were sent once daily automated SMS reminders to report their HIVST result for up to 7 days. | To assess feasibility and uptake of HIVST and linkage-to-care following HIVST and investigate the use of an SMS for participants to report their results to the study as well as a means for reminding participants of the need for confirmatory testing. | Use of an SMS to report HIVST results | Of the 428 participants who reported an HIVST result, 366 (85.5%) reported by SMS Of the 18 participants with newly-diagnosed HIV infection, six (33.3%) started ART within six months of HIVST, and additional two (11.1%) started ART more than six months after HIVST. |
| Baisley et al. 2019 | HIV Medicine | South Africa | Prospective cohort study - | 427 individuals from community aged ³ 15 years who were diagnosed HIV positive after having accepted to undertake an HIV home-based test | SMS reminders and nurse‐led telephone calls for those not linked within 1 month | To explore the efficacy of SMS reminders to individuals not in care 2 weeks after having been diagnosed with HIV during a survey | Linkage to care after undertaking an HIV home-based test | Only one third of individuals aged < 30 years has linked to care within 6 months. Early SMS reminders and telephone support for linkage are not sufficient to eliminate barriers to timely ART initiation among young adults, particularly young men |
| Georgette et al. 2017 | BMC Medical informatics and decision making | South Africa | Retrospective cohort study | 2255 adults living with HIV, receiving ART and enrolled in a SMS program adherence | Participants shall have a valid phone number to be included in the cohort. | To estimate rates of linkage to HIV care and antiretroviral treatment (ART) initiation after the introduction of home-based HIV counselling and testing (HBHCT) and telephone-facilitated support for linkage in rural South Africa. | HIV testing uptake and linkage to care in the first year of the program | SMS program was associated with an increase in prescription coverage when comparing the Exposed and Unexposed subgroups (AOR 1.29, 95% CI: 1.17–1.43, P < 0.001) |

**Economic studies**

| **First author and year** | **Journal** | **Location** | **Study design** | **Sample characteristics** | **Tool properties** | **Goal of the intervention** | **Outcomes** | **Results** |
| --- | --- | --- | --- | --- | --- | --- | --- | --- |
| George et al. 2018 | Plos One | Kenya | Costing analysis for 2 RCTs using SMS to raise awareness on HIVST availability at clinic | 2262 Truckers and 2196 female sex workers (FSW) | Not addressed | To explore strategic yet cost‐effective approaches to increase the uptake of HIV testing, especially among high‐risk populations | Cost-effectiveness of an mHealth intervention | Truckers: SMS cost (incl. staff time) per client tested was USD 9.93 in the intervention arm vs USD 21.25 in the standard of care arm FSW: SMS cost (incl. staff time) per client tested was USD 3.19 in the intervention arm vs USD 4.90 in the standard of care arm |
| Patel et al. 2017 | Medicine | Kenya | Comparison of SMS-based adherence interventions to standard-care using incremental cost-effectiveness ratios (ICER) + multivariate and univariate sensitivity analysis | Kenyan people living with HIV/AIDS initiating ART who own or have access to mobile phones and got enrolled in one of the two RCTs that were considered + AMPATH data base (multiyear cohort in East Africa) | Not addressed | To evaluate the cost-effectiveness of SMS-based adherence interventions and explore the added value of retention benefits. | Cost-effectiveness of a weekly SMS-based adherence intervention compared to usual care in people living with HIV/ AIDS initiating ART in Kenya | Weekly SMS-based interventions to support HIV treatment are very cost-effective by WHO standards in Kenya. The base case ICER for SMS interventions was $1037/QALY, which is below the WHO very cost-effective threshold of $US 2154/QALY. With additional retention benefits, the ICER improved to $864/QALY, making them even more efficient at extending QALYs. |
| Stevens et al. 2018 | Plos One | Swaziland | Cost simulation | PLHIV enrolled in the Link4health RCT which is a cluster randomized controlled trial performed from 2013 to 2015 in Swaziland that compared standard of care (N = 1,101) to the Link4Health combination strategy plus standard of care (N = 1,096) | Not addressed | To evaluate the impact and cost-effectiveness of a nationwide scale-up of the Link4Health strategy in Swaziland | Evaluation of total quality-adjusted life years (QALYs), incremental cost effectiveness ratios (ICERs) | The cost-effectiveness of the Link4Health strategy falls within the range observed in these single intervention studies, but not quite as cost effective as text messaging alone ($1,024/QALY [adjusted to USD2015]) if DALYs are considered a surrogate for QALYs. |
