# Supplementary material for: How mHealth Can Contribute to Improving the Continuum of Care: A Scoping Review Approach to the Case of Human Immunodeficiency Virus in Sub-Saharan Africa
Source: Public Health Rev. 2022 Sep 23;43:1604557. doi: 10.3389/phrs.2022.1604557 (PMC9537374; doi:10.3389/phrs.2022.1604557)
Supplement: Supplementary file 4 [file DataSheet1.docx]

***Supplementary file 1****: detailed search strategy (scoping review, Sub-Saharan Africa, 2017 – 2021).*

- (HIV OR AIDS) AND (testing OR test OR diagnostic OR “self-testing” OR “self-test” OR HIVST OR “home-based testing” OR HTC) AND (“linkage to care” OR linkage OR uptake) AND (mHealth OR “digital health” OR “mobile phone” OR SMS OR “text message”) AND Africa
- (HIV OR AIDS) AND (“treatment initiation” OR treatment OR ARV OR ART OR care) AND (“retention in care” OR retention OR uptake) AND (mHealth OR “digital health” OR “mobile phone” OR SMS OR “text message”) AND Africa
- (HIV OR AIDS) AND (“lab results” OR “turnaround time”) AND “viral load” AND (mHealth OR “digital health” OR “mobile phone” OR SMS OR “text message”) AND Africa
- (HIV OR AIDS) AND (“key populations” OR MSM OR “men who have sex with men” OR “sex workers”) AND (mHealth OR “digital health” OR “mobile phone” OR SMS OR “text message”) AND Africa
- (HIV OR AIDS) AND (“patient” OR “health provider” OR “health care provider” OR “health agent”) AND (mHealth OR “digital health” OR “mobile phone” OR SMS OR “text message”) AND Africa
